# Radical transfer in *E. coli* ribonucleotide reductase: a NH_2_Y_731_/R_411_A-α mutant unmasks a new conformation of the pathway residue 731†
†Electronic supplementary information (ESI) available. See DOI: 10.1039/c5sc03460d


**DOI:** 10.1039/c5sc03460d

**Published:** 2015-12-09

**Authors:** Müge Kasanmascheff, Wankyu Lee, Thomas U. Nick, JoAnne Stubbe, Marina Bennati

**Affiliations:** a Max Planck Institute for Biophysical Chemistry, 37077 Göttingen, Germany. Email: marina.bennati@mpibpc.mpg.de; b Department of Chemistry, University of Göttingen, 37077 Göttingen, Germany; c Department of Chemistry, Massachusetts Institute of Technology, Cambridge, Massachusetts 02139, USA. Email: stubbe@mit.edu

## Abstract

Ribonucleotide reductases (RNRs) catalyze the conversion of ribonucleotides to deoxyribonucleotides in all living organisms. The catalytic cycle of *E. coli* RNR involves a long-range proton-coupled electron transfer (PCET) from a tyrosyl radical (Y_122_˙) in subunit β2 to a cysteine (C_439_) in the active site of subunit α2, which subsequently initiates nucleotide reduction. This oxidation occurs over 35 Å and involves a specific pathway of redox active amino acids (Y_122_ ↔ [W_48_?] ↔ Y_356_ in β2 to Y_731_ ↔ Y_730_ ↔ C_439_ in α2). The mechanisms of the PCET steps at the interface of the α2β2 complex remain puzzling due to a lack of structural information for this region. Recently, DFT calculations on the 3-aminotyrosyl radical (NH_2_Y_731_˙)-α2 trapped by incubation of NH_2_Y_731_-α2/β2/CDP(substrate)/ATP(allosteric effector) suggested that R_411_-α2, a residue close to the α2β2 interface, interacts with NH_2_Y_731_˙ and accounts in part for its perturbed EPR parameters. To examine its role, we further modified NH_2_Y_731_-α2 with a R_411_A substitution. NH_2_Y_731_˙/R_411_A generated upon incubation of NH_2_Y_731_/R_411_A-α2/β2/CDP/ATP was investigated using multi-frequency (34, 94 and 263 GHz) EPR, 34 GHz pulsed electron–electron double resonance (PELDOR) and electron–nuclear double resonance (ENDOR) spectroscopies. The data indicate a large conformational change in NH_2_Y_731_˙/R_411_A relative to the NH_2_Y_731_˙ single mutant. Particularly, the inter-spin distance from NH_2_Y_731_˙/R_411_A in one αβ pair to Y_122_˙ in a second αβ pair decreases by 3 Å in the presence of the R_411_A mutation. This is the first experimental evidence for the flexibility of pathway residue Y_731_-α2 in an α2β2 complex and suggests a role for R_411_ in the stacked Y_731_/Y_730_ conformation involved in collinear PCET. Furthermore, NH_2_Y_731_˙/R_411_A serves as a probe of the PCET process across the subunit interface.

## Abstract 2

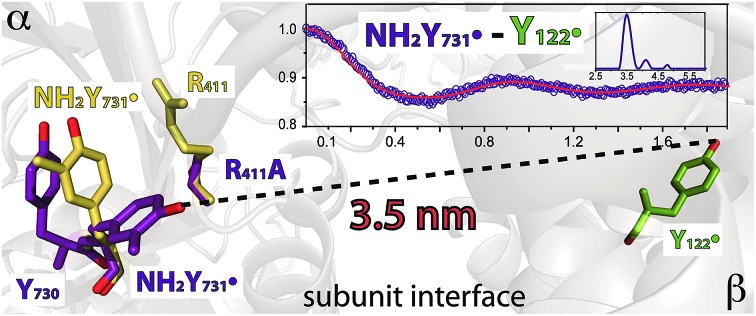
A new conformation of the *E. coli* RNR pathway residue 731 was trapped during long-range radical transfer across the αβ subunit interface.

## Introduction

Coupling of electron and proton transfers between donors and acceptors in proteins are ubiquitous in biology and can occur in a stepwise or concerted fashion. The concerted case avoids high energy intermediates and is designated as proton coupled electron transfer (PCET).^1^ The mechanisms of these couplings are fundamental to our understanding of photosynthesis, respiration, synthesis of DNA building blocks, and many other processes. Unresolved issues describing these mechanisms have been articulated in several recent comprehensive reviews, with different mechanisms dictated by transfer distances, protein environment and dynamics.^2^,^3^ When the proton and electron donor and acceptor are distinct, the mechanism involves orthogonal PCET; when the donor and acceptor are the same, it involves collinear PCET.^4^,^5^ A different mechanism in which a proton is transferred through water chains over long distances in concert with electron transfer (ET) has also been recently studied and discussed extensively in model systems.^6^,^7^ In all mechanistic cases, since the electrons and protons have very different masses, electrons tunnel over large distances (10–15 Å) while proton tunnelling is restricted to shorter distances, on the order of hydrogen bond lengths.^1^,^8^,^9^ This distance dependence complicates the issue of proton management. One important representative of the diversity of PCET mechanisms in proteins is found in the class I ribonucleotide reductases (RNRs). These enzymes catalyze the conversion of nucleotides to deoxynucleotides, the monomeric precursors required for DNA replication and repair in all eukaryotic and some prokaryotic organisms.^10^,^11^ In this paper, we use the *Escherichia coli* (*E. coli*) class Ia RNR as a model system to interrogate the PCET process across the interface of the two subunits of this enzyme, proposed to involve two redox active protein tyrosine residues, one on each subunit, and a water interface between the subunits.^12^

The *E. coli* RNR consists of two homodimeric subunits, α2 and β2.^13^ The enzyme is active when a transient α2β2 complex is formed.^14^ α2 contains the active site for nucleotide reduction and two allosteric effector binding sites that regulate the specificity and the rate of reduction.^15^–^19^ β2 harbors the essential di-iron tyrosyl radical cofactor (FeIII2–Y_122_˙).^20^,^21^ During each turnover, Y_122_˙-β2 oxidizes C_439_-α2 to a thiyl radical, which subsequently initiates dNDP production.^11^ There are X-ray structures of the individual subunits, and a docking model of the α2β2 complex places Y_122_˙ at a distance of about 35 Å from C_439_.^22^,^23^ These initial studies led to the first formulation of radical transfer (RT) in RNR *via* a radical hopping mechanism involving a pathway of conserved amino acids (Y_122_ ↔ [W_48_?] ↔ Y_356_ in β2 to Y_731_ ↔ Y_730_ ↔ C_439_ in α2). Biochemical^24^ and biophysical (EPR,^25^,^26^ SAXS,^27^ and cryoEM^28^) studies confirmed that the docking model provides a reasonable representation of *E. coli* RNR in its transient, active form and led to a detailed mechanism of RT over such a long distance.^4^,^14^,^24^ Nevertheless, in wild-type (wt) *E. coli* RNR, the rate limiting step, conformational change(s) upon substrate and allosteric effector binding to α2, has prevented spectroscopic detection of any intermediates in this process.^29^ The recent development of methods to site-specifically incorporate tyrosine analogs with altered p*K*_a_s and reduction potentials has permitted the detection of pathway radical intermediates^30^–^32^ and, combined with state-of-the-art EPR spectroscopy,^12^,^26^,^33^,^34^ has started to reveal the molecular basis of the long-range RT in RNR.^14^

**Fig. 1:**
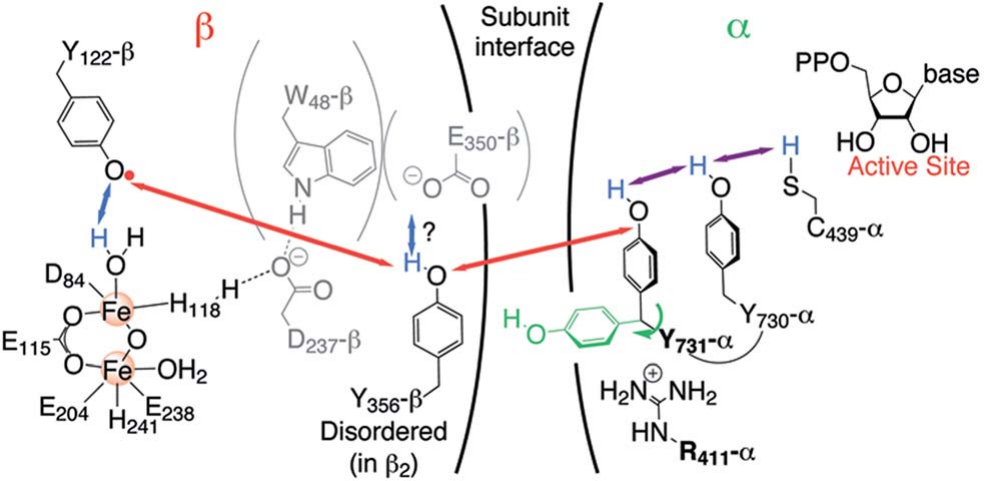
Current model of the long-range PCET in *E. coli* RNR. R_411_ is shown along with the redox active amino acid residues that are involved in this process (Y_122_ ↔ [W_48_?] ↔ Y_356_ in β2 ↔ Y_731_ ↔ Y_730_ ↔ C_439_ in α2). W_48_, D_237_ and E_350_ are shown in grey, because currently there is no evidence for their role in this process.^14^ The locations of Y_356_ and E_350_ are unknown, as they are within the flexible C-terminal tail (35 amino acids) of β2. Red, blue and purple arrows represent electron transfer, proton transfer and collinear PCET pathways, respectively.

These experiments have led to the current model illustrated in Fig. 1, which involves orthogonal PCET^35^ steps within subunit β2 and collinear PCET steps within the α2 subunit.^12^,^33^ However, the mechanism of the PCET process at the subunit interface between Y_356_ in β2 and Y_731_ in α2 remains elusive, as structural information on the C-terminal 35 amino acids of β2, including a putative proton acceptor E_350_ and Y_356_ (Fig. 1), is missing.^22^

Our recent high-field (HF) EPR/ENDOR and DFT investigations using the 3-aminotyrosine mutants NH_2_Y_730_-α2 and NH_2_Y_731_-α2, which generate the corresponding NH_2_Y˙ upon incubation with β2, CDP (substrate) and ATP (allosteric effector), established that an unusual stacked conformation of residues 730 and 731, observed in some X-ray structures of α2 (ref. 23 and 36) (see ESI, Fig. S1†), occurs in the α2β2 complex.^12^,^33^ However, the X-ray structure of NH_2_Y_730_-α2 (PDB ; 2XO4) alone exhibited multiple conformations for Y_731_-α2, with one rotated away from NH_2_Y_730_-α2 toward the α2β2 subunit interface.^30^ This “flipped” conformation was accompanied by reorientations of R_411_ and N_733_ in α2. Further comparison of NH_2_Y_730_˙-α2, NH_2_Y_731_˙-α2 and NH_2_Y_356_˙-β2 by HF EPR indicated that the electrostatic environment of all three transient NH_2_Y˙s is strongly perturbed and that their hydrogen bond interactions are intrinsically different.^12^,^33^ Interestingly, one of our DFT models of the protein environment for NH_2_Y_731_˙-α2 required R_411_-α2 to explain the perturbed *g*_*x*_ value observed and suggested that R_411_-α2 approaches to NH_2_Y_731_˙-α2 within 2.6 Å (Fig. S1†).^12^ Therefore, to examine the role of R_411_-α2 during the PCET process in *E. coli* RNR, we generated two mutants: R_411_A-α2 and the double mutant NH_2_Y_731_/R_411_A-α2. Here, we report the incubation of NH_2_Y_731_/R_411_A-α2 with β2/CDP and ATP, which generates the NH_2_Y_731_˙/R_411_A-α2β2 complex. Using advanced EPR methods, including 263 GHz pulse EPR and 34 GHz PELDOR/DEER (pulsed electron–electron double resonance) and ENDOR (electron–nuclear double resonance) spectroscopies, we have provided evidence for a new conformation of NH_2_Y_731_˙/R_411_ that is “flipped” towards the subunit interface in the α2β2 complex. This is the first time an alternative conformation of any pathway tyrosine (NH_2_Y_731_˙) has been observed and it provides a new probe of the PCET mechanism across the subunit interface, which remains unknown.

## Experimental

### Materials

4-(2-Hydroxyethyl)-1-piperazineethanesulfonic acid (Hepes) was purchased from EMD Bioscience. Adenosine-5′-triphosphate (ATP), cytidine-5′-diphosphate (CDP), reduced β-nicotinamide adenine dinucleotide phosphate (NADPH), hydroxyurea (HU), kanamycin (Km), chloramphenicol (Cm), 2XYT media, M9 Minimal Salts, l-arabinose (ara), β-mercaptoethanol (β-ME), streptomycin sulfate and NH_2_Y were purchased from Sigma-Aldrich. Isopropyl-β-d-thiogalactopyranoside (IPTG) and 1,4-dithiothreitol (DTT) were purchased from Promega. Tris(2-carboxyethyl)phosphine (TCEP) hydrochloride was purchased from Thermo Scientific. Nucleotide primers were purchased from Invitrogen, and Pfu Ultra II polymerase was purchased from Stratagene.

### Site-directed mutagenesis to generate R_411_A-α2 and NH_2_Y_731_/R_411_A-α2

The Quikchange kit (Stratagene) was used to generate each mutant according to the manufacturer's protocol. The templates pET28a-nrdA and pET28a-nrdA Y_731_Z^30^ were amplified with primer 5′-G CAG GAA CGT GCG TCT ACC GGT GCG ATC TAT ATT CAG AAC GTT GAC-3′ and its reverse complement and used to insert a GCG (Ala) at position 411. The sequences were confirmed by QuintaraBio Laboratory. All constructs contain an N-terminal (His)_6_-tag with a 10 amino acid linker.^30^

### Expression, purification and activity assays of R_411_A-α2 and NH_2_Y_731_/R_411_A-α2

(His)_6_-wt-α2 (2750 nmol min^–1^ mg^–1^) and wt-β2 (7000 nmol min^–1^ mg^–1^), and 1.2 Y˙/β2 were expressed and purified by standard protocols.^30^,^37^,^38^ All α2 mutants were pre-reduced with 30 mM DTT and 15 mM HU before use.^29^*E. coli* thioredoxin (TR, 40 U mg^–1^) and thioredoxin reductase (TRR, 1800 U mg^–1^) used in assays were isolated as previously described.^39^,^40^ (His)_6_-NH_2_Y_731_-α2 was purified as previously described.^30^ Expression and purification of R_411_A-α2 and NH_2_Y_731_/R_411_A-α2 followed previous protocols,^30^ except that the purification buffer (50 mM Tris, 5% glycerol, 1 mM PMSF, pH 7.6) for NH_2_Y_731_/R_411_A-α2 contained 1 mM TCEP. The yields of purified R_411_A-α2 and NH_2_Y_731_/R_411_A-α2 were 10–12 mg g^–1^ and 6–7 mg g^–1^ cell paste, respectively. The activity of R_411_A-α2 (0.2 μM) and NH_2_Y_731_/R_411_A-α2 (1 μM) was determined in the presence of 50-fold excess of wt-β2 with 3 mM ATP, 1 mM [^3^H]-CDP (4850 cpm nmol^–1^), 30 μM TR, 0.5 μM TRR, and 1 mM NADPH in assay buffer (50 mM HEPES, 1 mM EDTA, 15 mM MgSO_4_, pH 7.6). The amount of dCDP was determined by the method of Steeper and Steuart.^41^ For single turnover experiments, NH_2_Y_731_/R_411_A-α2 (5 μM) was incubated with wt-β2 (5 μM), 3 mM ATP, and 1 mM [^3^H]-CDP (20 000 cpm nmol^–1^) in assay buffer. The dissociation constant (*K*_d_) for R_411_A-α2 and wt-β2 was determined in H_2_O and D_2_O buffers by the competitive inhibition assay^42^ (SI-2, Fig. S2†).

### Samples for HF EPR and PELDOR spectroscopy

NH_2_Y_731_/R_411_A-α2 and wt-β2 were mixed 1 : 1 to a final concentration of 160–180 μM in D_2_O assay buffer as previously described.^32^,^34^ These protein concentrations resulted in >95% binding between subunits. The reaction was initiated at room temperature by adding CDP and ATP to final concentrations of 1 and 3 mM, respectively. The reactions were manually freeze-quenched in liquid N_2_ within 10–23 s. The PELDOR sample was prepared by adding glycerol-(OD)_3_ to a final concentration of 10% (v/v) 16 s after the initiation of the reaction. This reaction was manually freeze-quenched after 56 s as just described. The NH_2_Y_731_˙ accounted for 30–33% of the total spin for all the samples used in this work, which was similar to the yields reported previously.^30^,^32^

### HF pulsed EPR spectroscopy

Echo-detected (ESE: π/2 – *τ* – π – echo) EPR spectra at 263 GHz were recorded on a Bruker Elexsys E780 quasi optical spectrometer using a single mode (TE_011_) cylindrical resonator (E9501610 – Bruker BioSpin) with a typical quality factor of 500–1000. The maximum microwave power coupled to the resonator was about 15 mW. Samples for 263 GHz EPR were inserted in capillaries (0.33 mm OD, Vitrocom CV2033S) with typical volumes of *ca.* 50 nL. 94 GHz ESE spectra were recorded on a Bruker E680 spectrometer with a 400 mW W-band power setup (Bruker power upgrade – 2). Samples for 94 GHz ESE contained typical volumes of 2 μL in 0.84 mm OD capillaries (Wilmad S6X84). All manually freeze-quenched samples were immersed in liquid N_2_ and loaded into pre-cooled EPR cryostats.

### 34 GHz PELDOR spectroscopy

34 GHz ESE and PELDOR spectra were recorded on a Bruker E580 X/Q-band spectrometer equipped with a Bruker EN 5107D2 pulse EPR/ENDOR resonator. The spectrometer was power-upgraded with a Q-band TWT amplifier, providing about 170 W output power at 34.1 GHz. PELDOR experiments were recorded with an overcoupled resonator. The center of the mode was chosen for the pump frequency for measurements at 20 K. However, for measurements at 50 K the detection frequency was set in the center of the cavity mode to enhance detection sensitivity. Q-band samples contained typical volumes of 10 μL in 1.6 mm OD capillaries (Wilmad 222T-RB).

### Processing and simulation of EPR spectra

Spectra were processed by phasing and baseline correction. Derivatives of the absorption spectra were obtained by fitting every four points with a second order polynomial and differentiating the function in MATLAB_R2014b.^43^ EPR spectra were simulated using the EasySpin-4.5.5 “pepper”-routine which was run in MATLAB.^44^

### DFT calculations

DFT calculations were performed with the ORCA 3.0.0 program package.^45^ The geometry optimization of the neutral NH_2_Y˙ was performed using the unrestricted B3LYP^46^–^48^ hybrid density functional in combination with the def2-TZVPP basis set and def2-TZVPP/JK auxiliary basis set.^49^,^50^ To take into account the electrostatic environment of the radical intermediate at the protein interface, a solvation model (COSMO^51^) with the polarity of ethanol (*ε* = 24) was used. Otherwise, Grimme's dispersion correction^52^,^53^ and RIJCOSX^54^ approximations were employed. The energy converged to 10^–9^ E_h_. The hyperfine couplings and *g* values were calculated using NH_2_Y˙-C_4_ as the gauge origin.^55^,^56^ The def2-TZVPP basis set was consistent with the geometry optimization step.^50^ The C2–C1–Cβ–Cα dihedral angle of the NH_2_Y˙ was changed stepwise with a geometry optimization for each step. The *xyz* coordinates for one of the optimized models are given in the ESI.†

### PyMOL models

The docking model refers to the α2β2 complex structure generated from the individual wt-α2 and wt-β2 X-ray structures.^22^,^23^ In order to predict distances, the mutant *E. coli* RNR structure (PDB ; 2XO4)^30^ was overlaid with the wt-α2 structure in the docking model^23^ using PyMOL, which first performs a sequence alignment and then aligns the structures to minimize the root mean square deviation between the structures.

## Results and discussion

### Preparation and characterization of R_411_A-α2, NH_2_Y_731_/R_411_A-α2 and ND_2_Y_731_˙/R_411_A-α

Our recent studies on NH_2_Y_731_-α2 (ref. 12) suggested that R_411_ might interact with NH_2_Y_731_˙, partially accounting for the measured EPR and ENDOR parameters. To investigate this proposal, R_411_A-α2 was generated and characterized. Because the mutation is proposed to be at the interface of α2 and β2, the dissociation constant (*K*_d_) for subunit interactions was also examined and was determined to be 0.94 ± 0.33 μM (Fig. S2A†), ∼5 fold higher than that for wt-α2 (0.18 μM).^42^ Under these conditions, this mutant was shown to have a specific activity of 467 ± 22 nmol min^–1^ mg^–1^, 17% of that of the wt enzyme (2750 nmol min^–1^ mg^–1^). The reduced activity and weaker subunit binding suggest that R_411_ plays a functional role.

Furthermore, we characterized the role of R_411_ in the oxidation of Y_731_-α2 by generating the double mutant NH_2_Y_731_/R_411_A-α2. The *K*_d_ for subunit interactions between NH_2_Y_731_/R_411_A-α2 and wt-β2 was determined to be 8 ± 1 nM (Fig. S2C†), which is consistent with the formation of a tight complex when a NH_2_Y˙ is generated.^28^ Its specific activity was 13 ± 3 nmol min^–1^ mg^–1^, 0.4% of the specific activity of wt-RNR and in the range of contaminating wt-α2 activity.^32^ A more sensitive, one turnover assay was then employed to determine if this double mutant could generate any dCDP. When pre-reduced NH_2_Y_731_/R_411_A-α2 was mixed with wt-β2, CDP, and ATP for 5 min, only 0.036 ± 0.018 dCDP/α2 was observed, consistent with contaminating wt-α2. Thus, the double mutant is unable to make detectable dCDP, which is not unexpected, given the specific activities of the R_411_A and the NH_2_Y_731_-α2 mutants (see also SI-3 and Fig. S3†).

We next investigated whether NH_2_Y_731_˙ could be generated by NH_2_Y_731_/R_411_-α2, despite its inability to make dCDPs. NH_2_Y_731_/R_411_A-α2, wt-β2, CDP and ATP were studied by stopped-flow (SF) spectroscopy and the reaction was monitored at 320 nm, the absorption feature associated with the NH_2_Y˙ (Fig. S4,† red). The data were split into two time domains: 5 ms to 6 s and 25 s to 100 s. In the first time domain, NH_2_Y_731_˙ formation was fit to a double exponential with *k*_fast_ of 3.6 ± 0.5 s^–1^ (amplitude 8%) and *k*_slow_ of 0.47 ± 0.03 s^–1^ (amplitude 21%) (Table S1†). The rate constants for NH_2_Y_731_˙ in the single mutant control were similar: *k*_fast_ of 9.6 ± 0.6 s^–1^ and *k*_slow_ of 0.8 ± 0.1 s^–1^. However, in this case, the fast phase accounted for 27% and the slow phase accounted for 13% of the NH_2_Y_731_˙. The biphasic kinetics of NH_2_Y_731_˙ formation in both cases is attributed to multiple conformations that give rise to NH_2_Y_731_˙.^32^ From 25 s to 100 s, NH_2_Y_731_˙ in the double mutant reaction disappeared with a *k*_obs_ of 0.02 ± 0.003 s^–1^, while with the single mutant, disappearance occurred with a *k*_obs_ of 0.005 ± 0.002 s^–1^. Analysis of the Y_122_˙-β disappearance kinetics was unsuccessful at early time points due to the detection limits, as described in SI-4.

Given the distinct kinetics of our double mutant relative to the NH_2_Y_731_-α2, the 9 GHz EPR spectrum of the sample generated from the reaction of NH_2_Y_731_/R_411_A-α2 with wt-β2, ATP, and CDP quenched after 25 s was recorded and is shown in Fig. S5A and C.† Subsequent to subtraction of Y_122_˙, 32% of the total spin is associated with NH_2_Y_731_˙/R_411_A-α2 with no spin loss. This result is similar to that of the single mutant, NH_2_Y_731_˙.^30^,^32^ A comparison of their spectra, as shown in Fig. S5B,† revealed substantial differences in their hyperfine interactions, suggesting that further characterization of this radical might provide insight into the function of R_411_. Therefore, the role of R_411_ in the RT pathway was further studied with advanced EPR spectroscopy.

### HF EPR of ND_2_Y_731_˙/R_411_A-α2

**Fig. 2:**
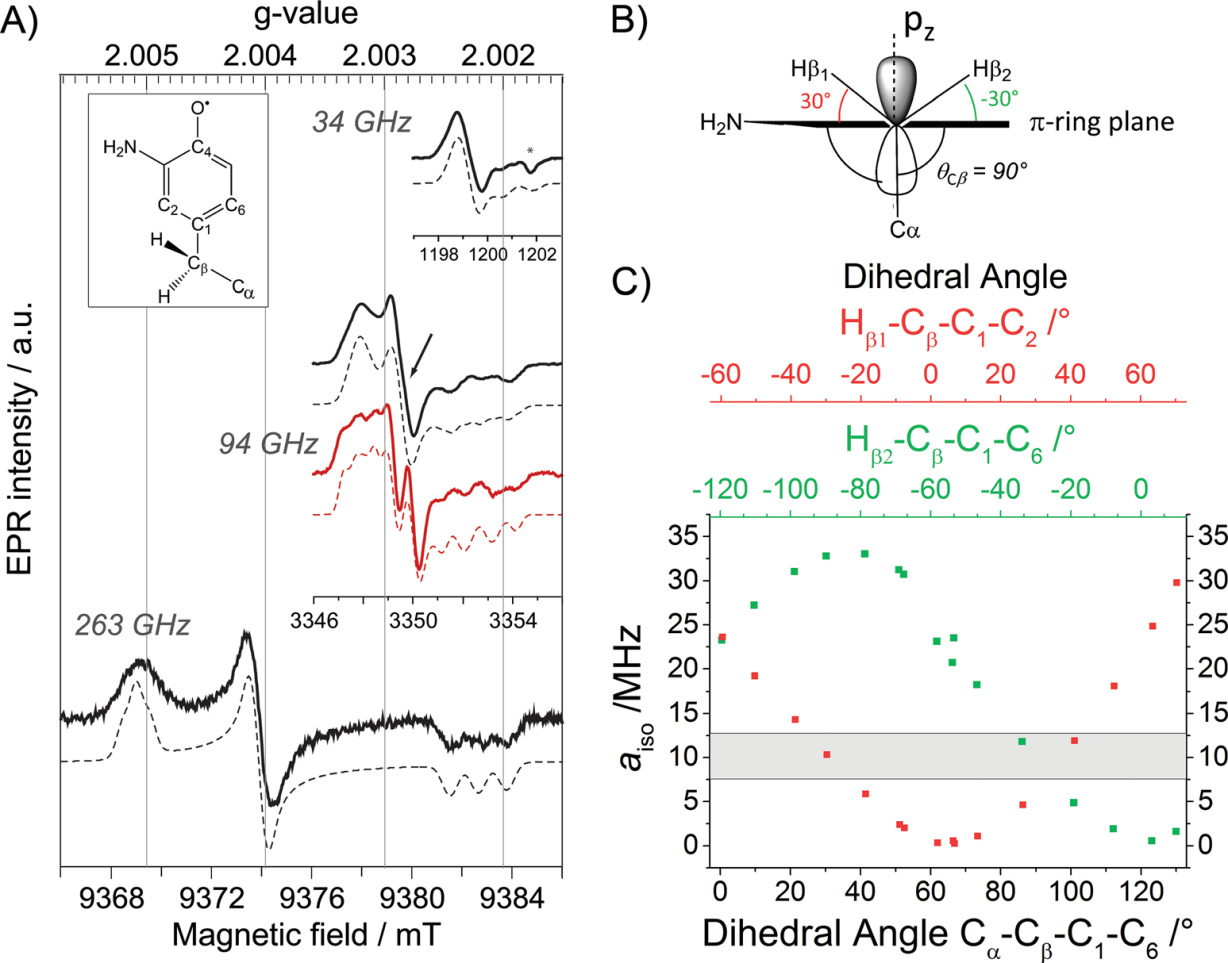
(A) Derivative EPR spectra (solid black lines) at 34 GHz (top), 94 GHz (middle) and 263 GHz (bottom) of ND_2_Y_731_˙/R_411_A-α2 with the corresponding simulations (dashed black lines). The 94 GHz EPR spectrum of ND_2_Y_731_˙-α2 in the single mutant (solid red line) with the corresponding simulation (dashed red line) is shown for comparison. The difference between the two spectra around *g*_*y*_ is marked with an arrow. The glass signal is marked with an asterisk. Exp. conditions (34 GHz): π/2 = 6 ns, *τ* = 280 ns, shot repetition time = 6 ms, shots/point = 80, number of scans = 10; (94 GHz): π/2 = 30 ns, *τ* = 280 ns, shot repetition time = 5 ms, shots/point = 100, number of scans = 50–100; (263 GHz): π/2 = 40 ns, *τ* = 270 ns, shot repetition time = 6 ms, shots/point = 250, number of scans = 36. Structure of NH_2_Y˙ is shown in the inset. (B) Orientation of the Cβ-methylene protons with respect to the phenol ring, as extracted from the observed hyperfine couplings. (C) *a*_iso_ as a function of the dihedral angle for each Cβ-methylene proton, calculated from a DFT model for NH_2_Y˙ (Fig. S6†).

To examine the generated ND_2_Y_731_˙/R_411_A-α2, we took advantage of the proximity of Y_122_˙ to the di-iron cluster and its altered relaxation properties. Pulsed EPR spectra of ND_2_Y_731_˙/R_411_A-α2 at 34, 94 and 263 GHz were recorded in D_2_O buffer at 70 K and are shown in Fig. 2A. The use of D_2_O considerably simplifies the EPR spectra due to the absence of ^1^H hyperfine (hf) splittings arising from the amino protons. The ND_2_Y_731_˙/R_411_A-α2 EPR spectrum at 34 GHz is mainly dominated by the large hf couplings with the deuterons of the amino group and the two Cβ-methylene protons.^34^ On the other hand, the 94 and 263 GHz EPR spectra are dominated by *g*-anisotropy, and the relative contributions of *g*- and hf-anisotropy are strongly dependent on the operating magnetic field. The *g* values of ND_2_Y_731_˙/R_411_A-α2 are best resolved at 263 GHz and are consistent with the values from our previous ND_2_Y˙ studies.^12^,^33^ The 94 GHz spectra reveal differences in the hf splitting of the Cβ-methylene protons (Fig. 2A, marked with an arrow): the large hf splitting of the Cβ-methylene proton visible in the central line of ND_2_Y_731_˙-α2 (red) is missing in ND_2_Y_731_˙/R_411_A-α2 (black). This splitting is also absent in the 263 GHz spectrum. The EPR spectra were simulated iteratively to find a global solution for the contributing hf couplings. All of the EPR data and simulations, in which the previously reported^34^ hf coupling for ^14^N is used, are consistent with the NH_2_Y_731_˙ generated in the NH_2_Y_731_˙/R_411_A-α2/β2 complex being a single, well-oriented radical species with one set of magnetic parameters, which are listed in Table 1 (see also Fig. S7†). This finding is not self-evident, as our previous experiments with other double mutants, NH_2_Y_731_˙/Y_730_F-α2 and NH_2_Y_730_˙/C_439_A-α2, showed distributions in *g* values indicative of multiple radical environments and/or molecular orientations.^12^

**Table 1:** Summary of *g* values and large hf couplings (>8 MHz) of ND_2_Y_731_˙ in the double and single mutants^*a*^

Sample	*g* values			*a*_iso_ (MHz)		
	*g*_*x*_	*g*_*y*_	*g*_*z*_	Hβ_1_	Hβ_2_	^14^N
ND_2_Y_731_˙/R_411_A-α	2.0051	2.0041	2.0022	10	10	12
ND_2_Y_731_˙-α^*b*^	2.0051	2.0041	2.0022	22	9	12

^*a*^Uncertainties in the *g* values and hf couplings are about 0.00005 and up to 10%, respectively, as obtained from the spectral simulations.

^*b*^ *g* values and hf couplings were reported in ref. 12 and 34, respectively.

Interestingly, we do not observe changes in the *g* values between ND_2_Y_731_˙/R_411_A-α and ND_2_Y_731_˙-α2. This is unexpected because the *g*_*x*_ value is affected by the electrostatic environment of a radical,^57^ and the R_411_A mutation has changed the local environment of ND_2_Y_731_˙, as demonstrated by the substantial changes in the Cβ-methylene ^1^H couplings (Table 1). These couplings are related to the dihedral angle *θ*_Cβ_ between the Cβ–H bond and the p_*z*_ orbital axis of C_1_ (Fig. 2B), and therefore provide information on the molecular orientation of the tyrosyl and 3-aminotyrosyl radicals.^34^ The dihedral angle can be extracted from the McConnell equation (*a*_iso(Cβ–H)_ = *B*_1_ × *ρ*_C1_ × cos^2^ *θ*_Cβ_),^58^ which provides a semi-empirical relationship for the observed isotropic constant *a*_iso_. The C2–C1–Cβ–Cα angle of ND_2_Y_731_˙/R_411_A-α2 is estimated to be ≈90° by using *B*_1_ of 162 MHz (ref. 59) for tyrosyl radicals, an electron spin density *ρ*_C1_ of 0.214,^12^ and an isotropic Cβ-methylene proton hf coupling *a*_iso_ = 10 ± 1 MHz (Table 1). This dihedral angle is indeed consistent with the hf couplings of the two Cβ-methylene ^1^H resonances being indistinguishable, as reported in Table 1 and seen in Fig. 2B and C. This result was confirmed by DFT calculations on the observed hf couplings of NH_2_Y˙, in which the ring orientation was modeled with respect to the backbone and showed a symmetric orientation relative to the p_*z*_ orbital axis of C_1_ (Fig. 2B). In this calculation, a *θ*_Cβ_ angle of 90° corresponds to *a*_iso_ = 9 ± 3 MHz (grey area in Fig. 2C) for both Cβ-methylene protons, H_β2/1_.

### ENDOR for detection of hydrogen bonds to ND_2_Y_731_˙/R_411_A-α2

**Fig. 3:**
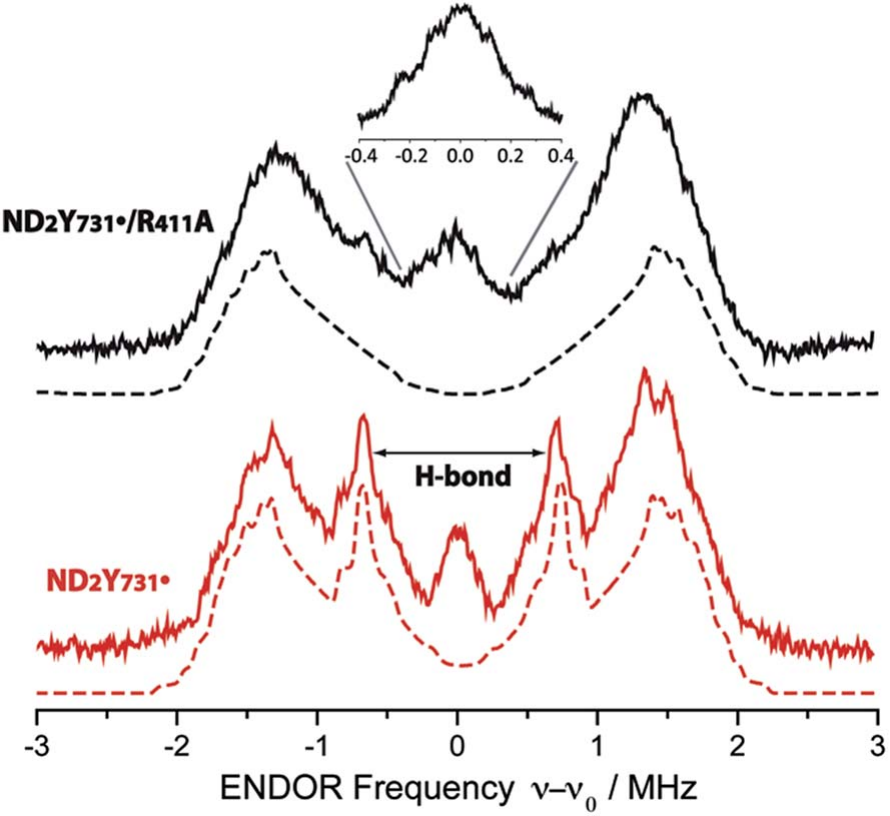
34 GHz ^2^H ENDOR spectra of ND_2_Y_731_˙/R_411_A-α2 (solid black line) and ND_2_Y_731_˙-α2 (solid red line). Simulations are shown with dashed lines. The hydrogen bond peaks, “H-bond”, assigned to a deuteron located between Y_730_-α2 and ND_2_Y_731_˙-α2 (ref. 12), are not observed for ND_2_Y_731_˙/R_411_A-α2. The inset shows the structured and broad matrix line of ND_2_Y_731_˙/R_411_A-α2. Exp. conditions: Mims ENDOR with π/2 = 6 ns, *τ* = 200 ns and 320 ns for the inset, shot repetition time = 15 ms, random RF acquisition^60^ with 1 shot/point, acquisition time = 15–20 h, *T* = 70 K. Excitation in the EPR line is set to *B*_0_||*g*_*y*_. ENDOR spectra are centered at the Larmor frequency of ^2^H, *ν*_0_ = 7.9 MHz at 1.2 T.

Given that the R_411_A mutation had little effect on *g*_*x*_, ^2^H ENDOR spectroscopy was used to further examine a possible correlation of the observed *g*_*x*_ value (*g*_*x*_ = 2.0051) with the hydrogen bonding environment. Fig. 3 illustrates the ^2^H Mims ENDOR spectra of ND_2_Y_731_˙-α2 and ND_2_Y_731_˙/R_411_A-α2. Both spectra contain a broad signal that extends over ±2 MHz, arising from the strongly coupled amino deuterons, which is a common feature of ND_2_Y˙ Mims ENDOR spectra.^12^,^33^ However, we observe that the ^2^H hf tensor previously assigned to the moderately strong hydrogen bond between Y_730_ and Y_731_ in ND_2_Y_731_˙-α2, which is almost perpendicular to the tyrosine ring plane,^12^ is absent in the ND_2_Y_731_˙/R_411_A-α2 spectrum. Therefore, the hydrogen bonding environment of NH_2_Y_731_˙/R_411_A-α2 is distinct from that of the single mutant, consistent with the different side chain conformations observed by HF EPR spectroscopy. Note that almost the complete EPR line of ND_2_Y_731_˙/R_411_A-α2 can be excited at 34 GHz by using very short microwave pulses, and thus hf couplings cannot be missed due to orientation selective effects.

Although no exchangeable moderately strong hydrogen bonds (*r*_O–H_ ∼ 1.7–2 Å) to ND_2_Y_731_˙/R_411_A-α2 are observed, the ENDOR spectrum of ND_2_Y_731_˙/R_411_A-α2 exhibits a broad and structured matrix line, which is associated with weak hf interactions of the radical with distant nuclei^61^ (see Fig. 3, inset). The structure in this matrix line suggests the presence of weakly coupled deuterons that cannot be resolved from the matrix ones (matrix line). We note that the ENDOR spectrum of ND_2_Y_731_˙/R_411_A-α2 is reminiscent of the one previously observed for ND_2_Y_356_˙-β2, also located at the subunit interface and likely surrounded by a defined hydrogen bonded network of water molecules.^12^ The similarity between the ENDOR spectra of ND_2_Y_356_˙-β2 and ND_2_Y_731_˙/R_411_A-α2 suggests a similar origin for the *g*_*x*_ values in these two mutants, which is distinct from that in ND_2_Y_731_˙-α2. As noted above, in the case of ND_2_Y_356_˙-β2 the *g*_*x*_ value was also strongly shifted (NH_2_Y_356_˙: *g*_*x*_ = 2.0049 *vs.* free NH_2_Y˙: *g*_*x*_ = 2.0061 (ref. 33)). Therefore, we propose that the *g*_*x*_-shift in NH_2_Y_731_˙/R_411_A-α2, as well as in ND_2_Y_356_˙-β2, arises from weakly coupled hydrogen bonds observed in the 0.3 MHz region of the ENDOR spectrum. The complexity of the *g* tensor interpretation was underlined by our recent DFT calculations, in which three distinct models for NH_2_Y_731_˙-α2 resulted in similar *g*-shifts.^12^ Overall, these data clearly indicate that the molecular orientation of ND_2_Y_731_˙/R_411_A-α2 is different to that of ND_2_Y_731_˙-α2 and is affected by R_411_A-α2 substitution.

### PELDOR gives evidence for a conformational change in ND_2_Y_731_˙/R_411_A-α2

**Fig. 4:**
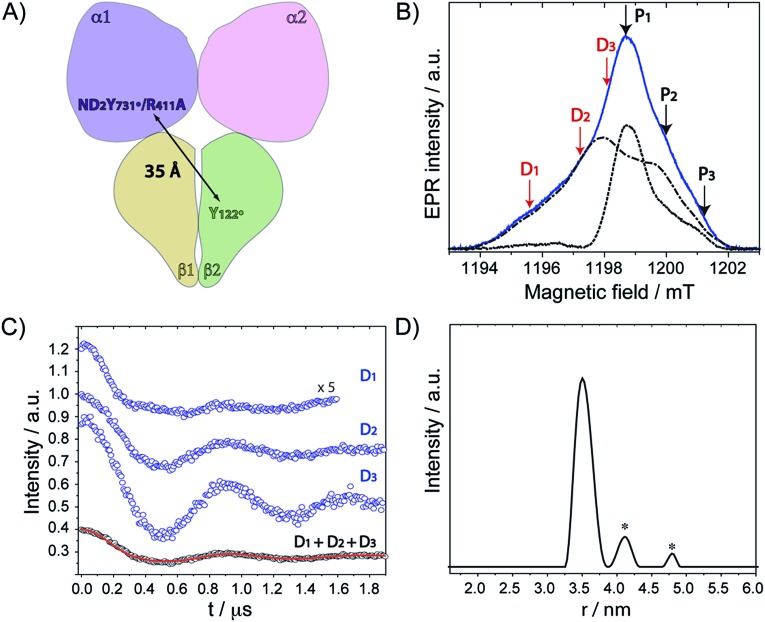
(A) Diagonal distance between Y_122_˙ in β2 and ND_2_Y_731_˙/R_411_A-α2. (B) The ESE spectrum of ND_2_Y_731_˙/R_411_A-α2 is composed of the Y_122_˙-β2 spectrum (dashed and dotted black line) and the ND_2_Y_731_˙-α2 spectrum (dotted black line). Detect (D) and pump (P) frequency positions for each PELDOR measurement are displayed by red and black arrows, respectively. Exp. conditions (EPR): π/2 = 6 ns, *τ* = 280 ns, shot repetition time = 120 ms, shots/point = 10, 4 scans, *T* = 20 K. (C) Background- and phase-corrected, normalized 34 GHz PELDOR time traces of three experimental setups (D_1_, D_2_, D_3_). The sum of the three traces (D_1_ + D_2_ + D_3_) was analyzed by DeerAnalysis 2015 (ref. 67) and is shown in black with the fitting overlaid in a solid red line. Detect/pump π pulse lengths for D_1_, D_2_ and D_3_ were 30 ns/12 ns, 30 ns/12 ns and 20 ns/14 ns, respectively. The frequency separation between detect and pump pulses was 80 MHz for all data sets. (D) Distance distribution obtained from the analysis in (C). Asterisks indicate artifacts attributed to the analysis procedure.

Our previous PELDOR studies^26^ have demonstrated that half sites reactivity of *E. coli* RNR allows for the detection of the diagonal inter-spin distance between Y_122_˙ in one αβ pair and any radical trapped in the second αβ pair (Fig. 4A).^25^,^62^ To gain insight into the location of NH_2_Y_731_˙/R_411_A-α2, three sets of PELDOR experiments were recorded using broadband excitation with a high-power Q-band set up at different excitation positions in the EPR line^63^–^66^ (see Fig. 4B and S8†). The recorded time traces are displayed in Fig. 4C and show substantial differences in modulation depth (10 to 50%), which is typical for orientation selection effects. Trace D1 also shows a higher frequency component that arises from the parallel component of a dipolar Pake pattern (Fig. S8†). For this reason, the background corrected PELDOR time traces from the three sets of experiments were summed and the resulting trace was analyzed as shown in Fig. 4C and D. Additional comparison of the Fourier-transformed traces (Fig. S8†) shows that the sum trace leads to an almost complete Pake pattern. Distance distribution analysis revealed a clear dominant peak at 35 Å with a distance distribution of Δ*r* = ±2.7 Å. We note that the error in the peak distance is much less than the distribution and is estimated to be ≤ ±0.5 Å. The width of the distance distribution is slightly larger than in previous measurements within the *E. coli* RNR α2β2 complex,^25^,^26^,^62^ suggesting more conformational heterogeneity for ND_2_Y_731_˙/R_411_A-α2, consistent with the observed flexibility of this residue. Nevertheless, the results clearly indicate that the R_411_ mutation induces a conformational change of ND_2_Y_731_˙ into a new well-defined conformation.

The peak distance of 35.0 Å has never been observed between any radicals formed in this pathway before, and it is 3 Å shorter than that previously measured for ND_2_Y_731_˙-α2.^26^ This distance might appear to be rather close to the initial distance (prior to turnover) between the two stable Y_122_˙s, that is 33.1 ± 0.2 Å.^62^ To confirm our assignment, we recorded PELDOR experiments at higher temperature (50 K), in which the Y_122_˙-β2 contribution to the re-focused echo is filtered and ND_2_Y_731_˙-α2 is the only radical species detected (Fig. S9†). However, Y_122_˙-β2 can still be excited by the pump pulse and contributes to the PELDOR signal. Under these conditions, any distance observed in the PELDOR experiments at 50 K is related to Y_122_˙–ND_2_Y_731_˙ and cannot be associated with the Y_122_˙–Y_122_˙ distance, as the latter radical is not detected. The distance distribution analysis of the 50 K measurements yielded a peak distance of 35.3 Å with a distribution of Δ*r* = ± 2.0 Å, and thus validated our assignment (see Fig. S9†).

**Fig. 5:**
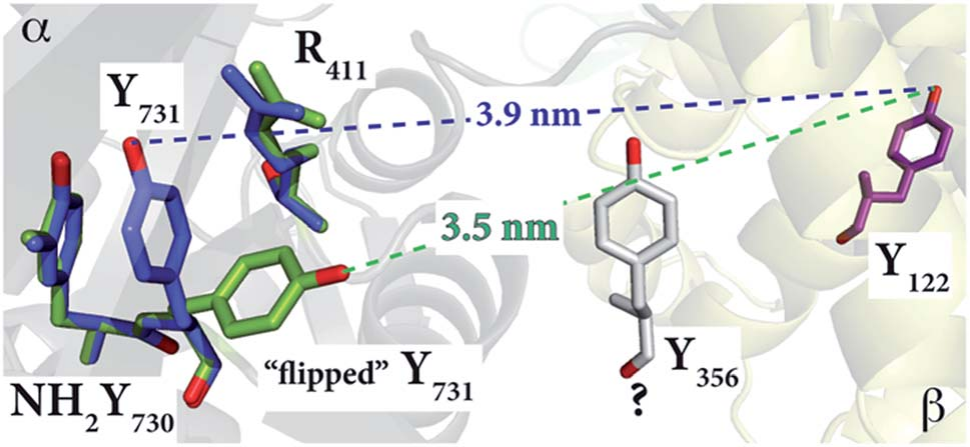
The *E. coli* Y_730_NH_2_Y-α2 structure (; 2XO4)^30^ in green shows the reoriented Y_731_ overlaid with the stacked Y_731_ in a different monomer (blue) of the unit cell. The diagonal distances between the “flipped” and non-flipped Y_731_ and Y_122_ are 3.5 nm and 3.9 nm, respectively. These distances, which are between two phenolic oxygen atoms of the tyrosine residues, are based on the alignment with the *E. coli* α2β2 docking model. Residue Y_356_ is shown in grey with a “?” because its position is unknown.

To gain more insight into the conformation of NH_2_Y_731_˙/R_411_A-α2 and the role of R_411_, we examined the available X-ray structures of *E. coli* α2s in the R_411_ region. In the structure of *E. coli* NH_2_Y_730_-α2 (; 2XO4),^30^ Y_731_ is flipped away from NH_2_Y_730_, as shown in Fig. 5. This altered conformation is compared with a second α in the unit cell, in which the Y_731_ is not flipped. To match the 35 Å distance observed by PELDOR spectroscopy, the aromatic ring of NH_2_Y_731_ must rotate away from Y_730_ toward the β2 subunit, as observed for Y_731_ in the *E. coli* Y_730_NH_2_Y-α2 structure (Fig. 5).

This reorientation is also supported by the ENDOR data, which indicate that the stacked conformation between NH_2_Y_731_˙ and Y_730_ with a shared, perpendicular hydrogen bond is absent in NH_2_Y_731_˙/R_411_A-α2, and that the radical is instead surrounded by weakly coupled hydrogen bonds, likely water molecules at the α2β2 subunit interface. The exposure of NH_2_Y_731_˙/R_411_A-α2 to the interface and the buffer in this new conformation might be the origin of the instability of the radical as compared to the single mutant (Table S1†).

We have also examined another possible conformation, in which the amino group of NH_2_Y_731_-α2 moves to occupy the vacancy created by the mutation of arginine to alanine. This conformation is displayed in Fig. S10.† However, in this case the expected distance between the oxygen atoms of NH_2_Y_731_ and Y_122_ exceeds the observed distance by ≥2 Å. We note that the “flipped” conformation has not been observed in the single mutant NH_2_Y_731_-α2 or in the double mutant NH_2_Y_731_/Y_730_F-α2, in which Y_731_ lacks its hydrogen bonding partner,^12^ suggesting the importance of R_411_ in stabilizing the stacked conformation. This change between a flipped and non-flipped conformation of the interface Y might play an active role in the PCET process between Y_731_ and Y_356_ in wt RNR, the mechanism of which is still not understood. With the wt enzyme, this conformational change is kinetically masked by physical gating, which rate-limits RNR, and is too fast to be detected based on the recently measured rate constants for electron transfer (ET) (10^4^ to 10^5^ s^–1^) at the interface by photo-RNRs that unmask this gating.^68^,^69^ Thus, the R_411_A mutation might have fortuitously allowed detection of this movement at the subunit interface.

While the lack of structural information at the subunit interface poses a challenge for a mechanistic understanding of interfacial PCET, the detection of the NH_2_Y_731_˙/R_411_ provides us with a spectroscopic probe of this interface. Mutagenesis and site-specific isotopic labeling of interface residues could provide us with additional insight into how this step is controlled. Finally, the mechanism of PCET across the subunit interface observed with the *E. coli* RNR is likely to be conserved in all class I RNRs based on their subunit structures and the conserved weak subunit associations dictated by the C-terminal tail of β2.^70^,^71^ The pathway for oxidation is conserved between RNR classes Ia, Ib and Ic, as is the regulation of the pathway by NDP/effector binding.^72^ Thus, while the “details” of the radical transfer mechanism might be different in the individual class I RNRs, general principles will likely emerge from the studies on *E. coli* RNR, given all of the evolutionarily conserved features.

## Conclusions

This study has revealed that the *E. coli* RNR double mutant NH_2_Y_731_/R_411_A-α2 unmasks a new conformation of pathway residue 731 in the α2β2 complex. This is the first experimental evidence for the flexibility of this pathway or any pathway residue in the active enzyme. The results have provided insight into the mechanisms of PCET within α2, as well as through the α2β2 interface. First, R_411_ appears to play a role in the stabilization of the stacked conformation of Y_731_ and Y_730_, and thus in the facilitation of collinear PCET within the α2 subunit. Second, the new conformation is consistent with Y_731_ pointing toward the subunit interface, in the direction of the adjacent pathway residue Y_356_, located in the flexible C-terminal tail of subunit β2. The flexibility of these two contiguous pathway residues, which have been suggested to communicate during PCET,^69^ might be the key to driving the RT chemistry at the subunit interface through water clusters.^6^,^7^ This opens up a new hypothesis for the PCET mechanism between residues Y_731_–α2 and Y_356_–β2, which could involve a gated conformational change in Y_731_–α2 in wt RNR on a fast time scale, not observable without the R_411_A mutation. While this hypothesis remains to be proven, the present results will serve as a basis to design new experiments aimed at detecting a possible combined role of Y_731_–α2 and Y_356_–β2 in PCET through the subunit surface.

## Supplementary Material

Supplementary information
