# Supplementary material for: Radical transfer in E. coli ribonucleotide reductase: a NH2Y731/R411A-α mutant unmasks a new conformation of the pathway residue 731
Source: Chem Sci. 2015 Dec 9;7(3):2170–8. doi: 10.1039/c5sc03460d (PMC5968753; doi:10.1039/c5sc03460d)
Supplement: Supplementary file 1 [file SC-007-C5SC03460D-s001.pdf]

Electronic Supplementary Material (ESI) for Chemical Science.
This journal is © The Royal Society of Chemistry 2015

Supporting Information

Radical Transfer in *E. coli* Ribonucleotide Reductase: A $NH_2Y_{731}/R_{411}A$-α mutant unmasks a new conformation of the pathway residue 731.

Müge Kasanmascheff [a, b], Wankyu Lee [c], Thomas U. Nick [a], JoAnne Stubbe [c] and Marina Bennati [a, b]

[a] Max Planck Institute for Biophysical Chemistry, 37077 Göttingen, Germany
[b] Department of Chemistry, University of Göttingen, 37077 Göttingen, Germany
[c] Department of Chemistry, Massachusetts Institute of Technology, Cambridge, Massachusetts 02139, United States

Figure S1. The unusual stacked conformation of Y_{731} and Y_{730} in wt-α2 and a current DFT model of the NH_2Y_{731}•-α2/wt-β2 complex.

SI-2: K_d determination of $R_{411}A$-α2 or $NH_2Y_{731}/R_{411}A$-α2 with wt-β2.

Figure S2. K_d determination for $R_{411}A$-α2/wt-β2 and $NH_2Y_{731}/R_{411}A$-α2/wt-β2 determined by the competitive inhibition assay.

SI-3: Reaction of $R_{411}A$-α2 or $NH_2Y_{731}/R_{411}A$-α2, wt-β2, CDP and ATP monitored by 9 GHz EPR spectroscopy.

Figure S3: 9 GHz CW-EPR spectrum of $R_{411}A$-α2 or $NH_2Y_{731}/R_{411}A$-α2 with wt-β2, N_3CDP and ATP.

SI-4: Reaction of $NH_2Y_{731}/R_{411}A$-α2, wt-β2, CDP and ATP monitored by stopped-flow (SF) Vis spectroscopy.

Table S1: Kinetics of NH_2Y• formation and disappearance for NH_2Y_{731}•/$R_{411}A$-α2 and NH_2Y_{731}•-α.

Figure S4: Reaction of $NH_2Y_{731}/R_{411}A$-α2, wt-β2, CDP and ATP by SF Vis spectroscopy.

SI-5: Monitoring the reaction of $NH_2Y_{731}/R_{411}A$-α2 with wt-β2 by 9 GHz EPR spectroscopy.

Figure S5: 9 GHz CW-EPR spectrum of $NH_2Y_{731}/R_{411}A$-α2, wt-β2, CDP, ATP and comparison of the NH_2Y_{731}•/$R_{411}A$-α and NH_2Y_{731}•-α 9 GHz EPR spectra.

Figure S6: Conformations of $NH_2Y\bullet$ side chain.

Table S2: Sample *xyz* coordinates (Å) of an optimized model.

Figure S7: Comparison of simulation vs. experimental data for the 94 GHz EPR spectrum of $ND_2Y_{731}\bullet/R_{411}A$-$\alpha$.

Figure S8: PELDOR excitation of $ND_2Y_{731}\bullet/R_{411}A$-$\alpha$ measured at 20 K and 34 GHz.

Figure S9: PELDOR measurement with $ND_2Y_{731}\bullet/R_{411}A$-$\alpha$ at 50 K.

Figure S10: Examination of another possible conformation for $ND_2Y_{731}\bullet/R_{411}A$-$\alpha$

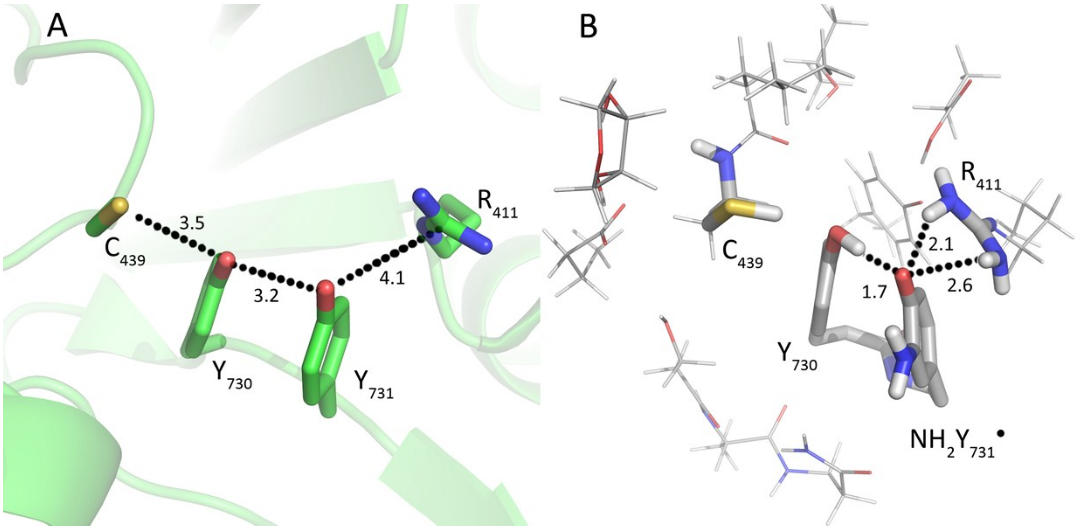

Figure S1. **A)** The unusual stacked conformation of Y_{731} and Y_{730} in wt-α2 as observed in the crystal structure (PDB 2X0X)[1]. **B)** DFT optimized structure of NH_2Y_{731}•-α2.[2] R_{411} is observed in close contact to NH_2Y_{731}•-α2, if no water molecules were considered in the simulation. Distances are given in Ångström.

SI-2: K_d determination of $R_{411}A$-$\alpha 2$ or $NH_2Y_{731}/R_{411}A$-$\alpha 2$ with wt-$\beta 2$. Because the mutation is proposed to be at the interface of $\alpha 2$ and $\beta 2$, we investigated the K_d for subunit interactions and determined it to be 0.94 ± 0.33 μM (Fig. S2A), which is ~5 fold higher than wt-$\alpha 2$ (0.18 μM).[3] The same experiment was repeated in D_2O assay buffer (K_d = 0.90 ± 0.19 μM, Fig. S2B) to ensure binding remains the same in the conditions of the EPR experiments. For $NH_2Y_{731}/R_{411}A$-$\alpha 2$, the K_d was determined to be 8 ± 1 nM, which is consistent with formation of a tight complex when a $NH_2Y\bullet$ is generated, described previously.[4] The K_d was determined by the spectrophotometric competitive inhibition assay with $R_{411}A$-$\alpha 2$ or $NH_2Y_{731}/R_{411}A$-$\alpha 2$ and wt-$\beta 2$.[3] The $R_{411}A$-$\alpha 2$ or $NH_2Y_{731}/R_{411}A$-$\alpha 2$ was used as the inhibitor, and concentrations varied between 10 nM and 5 μM. The relative activities in the presence of inhibitor were used to calculate a K_d with equation 1. $[I\bullet\beta 2]_{bound}$ is the concentration of inhibitor complexed with wt-$\beta 2$ and $[I]_{free}$ is the concentration of unbound inhibitor.[3]

$$[I\bullet\beta 2]_{bound} = \frac{[I]_{free}}{(K_d + [I]_{free})} \qquad \text{eq. 1}$$

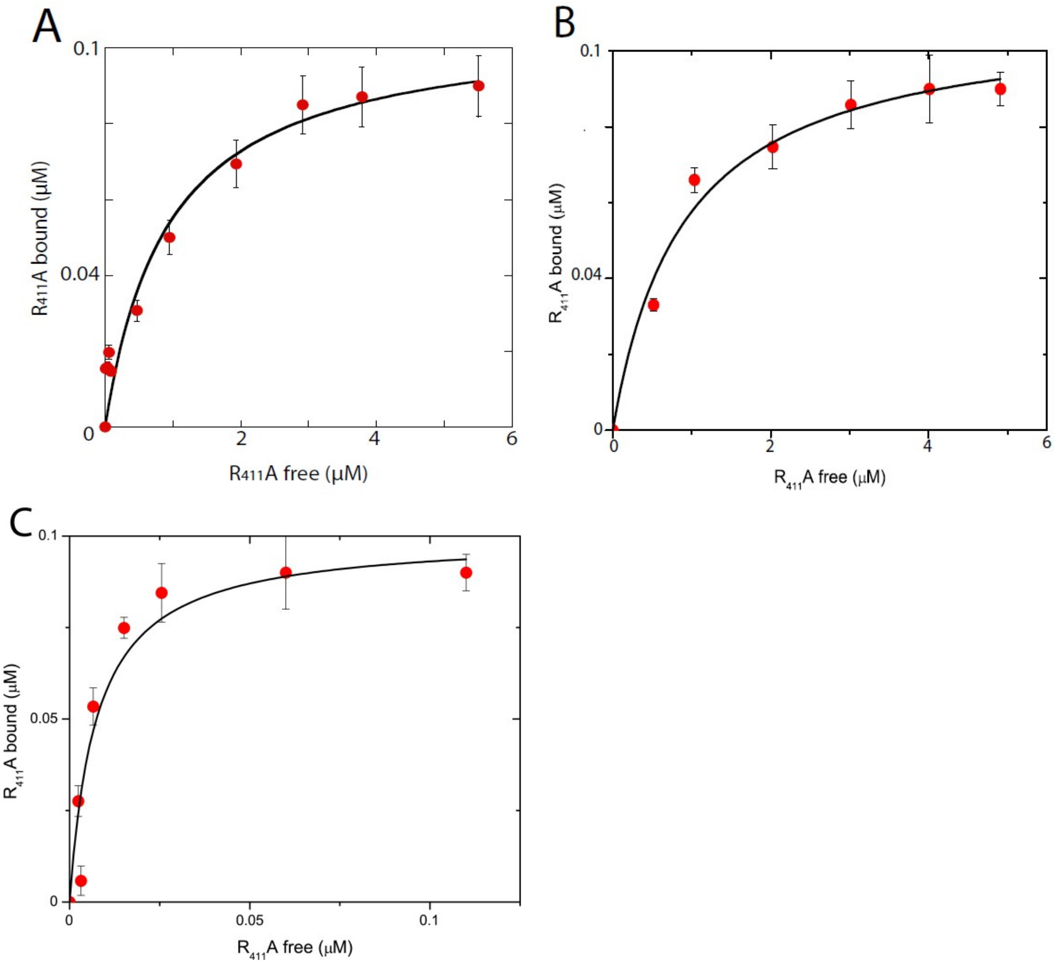

Figure S2. **A)** The K_d for the R_{411}A-α2/ wt-β2 complex determined by the competitive inhibition assay in the presence of CDP and ATP in H_2O assay buffer. The data shown (red) are an average of two replicates, with error between measurements <10%. The data were fit (solid black line) to the standard K_d equation (eq. 1) to yield 0.94 ± 0.33 µM. **B)** The K_d for the R_{411}A-α2/ wt-β2 complex determined by the competitive inhibition assay in D_2O assay buffer. The average of two replicates (red) are shown, with error between measurements <10%. The data were fit (solid black line) to the standard K_d equation (eq. 1) to yield 0.90 ± 0.19 µM. **C)** The K_d for the NH_2Y_{731}/R_{411}A-α2/ wt-β2 complex determined by the competitive inhibition assay in the presence of CDP and ATP in H_2O assay buffer. The data shown (red) are an average of two replicates, with error between measurements <10%. The data were fit (solid black line) to the standard K_d equation (eq. 1) to yield 8 ± 1 nM.

SI-3: Reaction of R_{411}A-α2 or NH_2Y_{731}/R_{411}A-α2, wt-β2, N_3CDP and ATP monitored by 9 GHz EPR spectroscopy. The 2`-azido-2`-deoxycytidine 5`-diphosphate (N_3CDP) is a stoichiometric inhibitor that needs C_{439}• mediated hydrogen atom abstraction from the substrate for inactivation. Reactions with this inhibitor form nitrogen centered radical (N•) covalently bound in the active site, and full inactivation of wt-RNR yields 50% N• from Y_{122}•, consistent with previous studies.[5, 6] The reaction mixture in a final volume of 240 µL contained 30 µM R_{411}A-α2 or NH_2Y_{731}/R_{411}A-α2, 30 µM wt-β2 (1.2 Y•/β2), 3 mM ATP, 0.15 mM N_3CDP and was initiated by addition of the mutant α2. The reaction was aged for 1 min at 25 ^{o}C and then quenched in liquid isopentane. EPR spectra were obtained with a Bruker EMX X-band (9 GHz) spectrometer equipped with a quartz finger dewar filled with liquid N_2 at 77 K at the MIT Department of Chemistry Instrumentation Facility (DCIF). The EPR parameters were: microwave frequency, 9.34 GHz; power, 100 µW; modulation amplitude, 1.5 G; modulation frequency, 100 kHz; time constant, 5.12 ms; scan time, 41.9 s. The reaction with R_{411}A-α2 exhibited 2% spin loss. Quantitation of N•-α2 and Y_{122}•-β2 was 51 ± 3% and 47 ± 3%, respectively, of total initial spin. This result is consistent with R_{411}A-α2 being active. The reaction with NH_2Y_{731}/R_{411}A-α2 contains Y_{122}•-β2 and no N•-α2. Overall spin loss was 47 ± 2%, which is consistent with expected quenching of NH_2Y• and Y_{122}• observed by SF-vis spectroscopy. This result is consistent with NH_2Y_{731}/R_{411}A-α2 being inactive in dCDP formation.

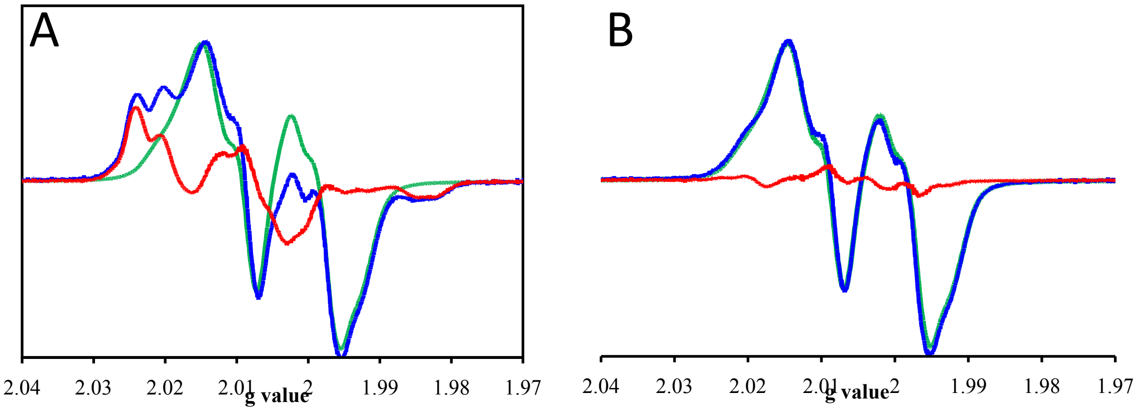

Figure S3: Reaction of 30 µM R_{411}A-α2 or 30 µM NH_2Y_{731}/R_{411}A-α2 with 30 µM wt-β2, 0.15 mM N_3CDP and 3 mM ATP quenched after 1 min in a liquid isopentane bath and then monitored by 9 GHz EPR spectroscopy. **A)** The EPR spectrum of the reaction mixture with R_{411}A-α2 contains Y_{122}•-β2 and N•-α2. The reference Y_{122}•-β2 (green) spectrum was subtracted from the composite spectrum (blue) to yield the N•-α2 (red) spectrum. The reaction exhibited 2% spin loss. Quantitation of N•-α2 was 51 ± 3%

and remaining Y_{122}•-β2 was 47 ± 3% of total initial spin. **B)** The EPR spectrum of the reaction mixture with NH_2Y_{731}/R_{411}A-α2 contains Y_{122}•-β2. No detectable N•-α2 in the composite spectrum (blue) prompted subtraction by Y_{122}•-β2 (green) to yield minor radical feature differences for Y_{122}•-β2 upon initiation (red). Overall spin loss was 47 ± 2%, which is consistent with expected quenching of NH_2Y• and Y_{122}• observed by SF-vis spectroscopy.

SI-4: Reaction of NH_2Y_{731}/$R_{411}A$-α2, wt-β2, CDP and ATP monitored by SF Vis spectroscopy. SF kinetics were performed on an Applied Photophysics DX. 17MV instrument equipped with the Pro-Data upgrade. All reactions were carried out in assay buffer maintained at 25 ^{o}C by Lauda water bath circulation. The contents of one syringe containing pre-reduced NH_2Y_{731}/$R_{411}A$-α2 and ATP in assay buffer were rapidly mixed with the contents of the second syringe containing wt-β2 and CDP to give final concentrations of 5 μM NH_2Y_{731}/$R_{411}A$-α2/wt-β2, 3 mM ATP and 1 mM CDP. The reaction was monitored at 320 nm for NH_2Y_{731}•/$R_{411}A$-α2 (ε ~ 11,000 $M^{-1}cm^{-1}$) using PMT detection. Efforts to monitor loss of Y_{122}•-β2 at 410 nm for (ε = 3700 $M^{-1}cm^{-1}$) were unsuccessful at early time points due to poor signal to noise. The results represent the average of 5 spectra, and fits were calculated with OriginPro software to minimize residuals. Equation 2 was used for double exponential fitting, where y_0 is a constant, A_1 and A_2 represent the amplitudes and R_1 and R_2 represent the rate constants of the two phases. Equation 3 was used for single exponential fitting.

$$y = y_0 + A_1 e^{-R_1 x} + A_2 e^{-R_2 x} \qquad \text{eq.2}$$

$$y = y_0 + A_1 e^{-R_1 x} \qquad \text{eq. 3}$$

The kinetics of NH_2Y_{731}•/$R_{411}A$-α formation were studied by SF Vis spectroscopy. NH_2Y_{731}•/$R_{411}A$-α formation (320 nm, red line Figure S4) was plotted as an average of 5 trials and was fit by a double exponential (black line Figure S4). A poor fit due to complicated kinetics prompted re-fitting by breaking the trace into two portions: NH_2Y_{731}•/$R_{411}A$-α formation and subsequent NH_2Y_{731}•/$R_{411}A$-α quenching. NH_2Y_{731}•/$R_{411}A$-α formation fitted to a double exponential equation with a fast phase of 3.6 ± 0.5 s^{-1}, 8% amplitude change and a slow phase of 0.47 ± 0.05 s^{-1}, 21% amplitude change. The radical quenching fit to a single exponential with a rate constant of 0.018 ± 0.003 s^{-1}, 23% radical lost. The kinetic rate constants are summarized, Table S1. Each kinetic phase of NH_2Y_{731}•/$R_{411}A$-α formation should correspond to those of Y_{122}•-β loss. An 8% amplitude change for Y_{122}•-β disappearance (410 nm), using an extinction coefficient of 3700 $M^{-1}cm^{-1}$, is a ΔOD of 0.002, too small of a change to accurately fit given the amount of replicates we could perform. We were therefore unable to make this measurement.

Table S1. Kinetics of $NH_2Y\bullet$ formation and quenching for $Y_{731}NH_2Y\bullet/R_{411}A$-*α* and $Y_{731}NH_2Y\bullet$-*α*.

	**$NH_2Y\bullet$ formation/quenching**					
**Mutant**	k_1 (s^{-1})	$\%A_1$	k_2 (s^{-1})	$\%A_2$	k_3 (s^{-1})	$\%A_3$
**NH_2Y_{731}/ $R_{411}A$-*α*[a]**	3.6 ± 0.5	8 ± 1	0.47 ± 0.03	21 ± 2	-0.02 ± 0.003	-23 ± 2
**NH_2Y_{731}-*α*[b]**	9.6 ± 0.6	27 ± 2	0.8 ± 0.1	13 ± 1	-0.005± 0.002	-21 ± 2

a) The kinetic parameters were obtained from a double exponential fit to 5 traces on the 5 ms to 6 s region and a single exponential fit to the 25 s to 100 s region. b) Same as in a) except 5 µM NH_2Y_{731}-*α*2 was used.

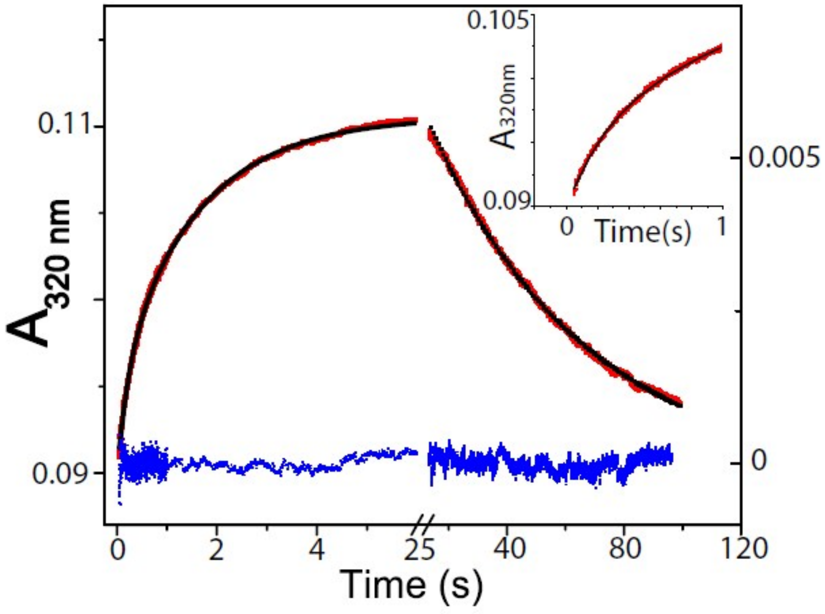

Figure S4: The kinetics of $NH_2Y_{731}\bullet/R_{411}A$ formation (320 nm, red) for the reaction of 5 µM $NH_2Y_{731}/R_{411}A$-*α*2, 5 µM wt-*β*2, CDP/ATP (1 mM/3 mM). Note the x axis is split into two time domains: 0 to 6 s and 25 s to 100s. Double exponential fits to the data are shown in black and the residuals are in blue. (*Inset*) Expanded view of the first 1 s of $NH_2Y_{731}\bullet/R_{411}A$ (320 nm, red) with the fit to the data shown in black.

SI-5: Reaction of NH_2Y_{731}/R_{411}A-α2, wt-β2, CDP and ATP monitored by 9 GHz EPR spectroscopy. The reaction mixture in a final volume of 300 µL contained 30 µM NH_2Y_{731}/R_{411}A-α2, 30 µM wt-β2 **(**1.2 Y•/β2**)**, 3 mM ATP, 1 mM CDP in assay buffer and was initiated by addition of the mutant α2. The reaction was aged for 25 s at 25 ^{o}C and then quenched in liquid isopentane. EPR spectra were obtained as described in SI-3. WinEPR (Bruker) was used for EPR spin quantitation by measuring the normalized double integral intensity ($\frac{DI}{N}$) of the spectrum, and correcting for power, number of scans, and modulation amplitude, and comparing the $\frac{DI}{N_C}$ to that of a wt-β2 standard with a known [Y_{122}•].[7]

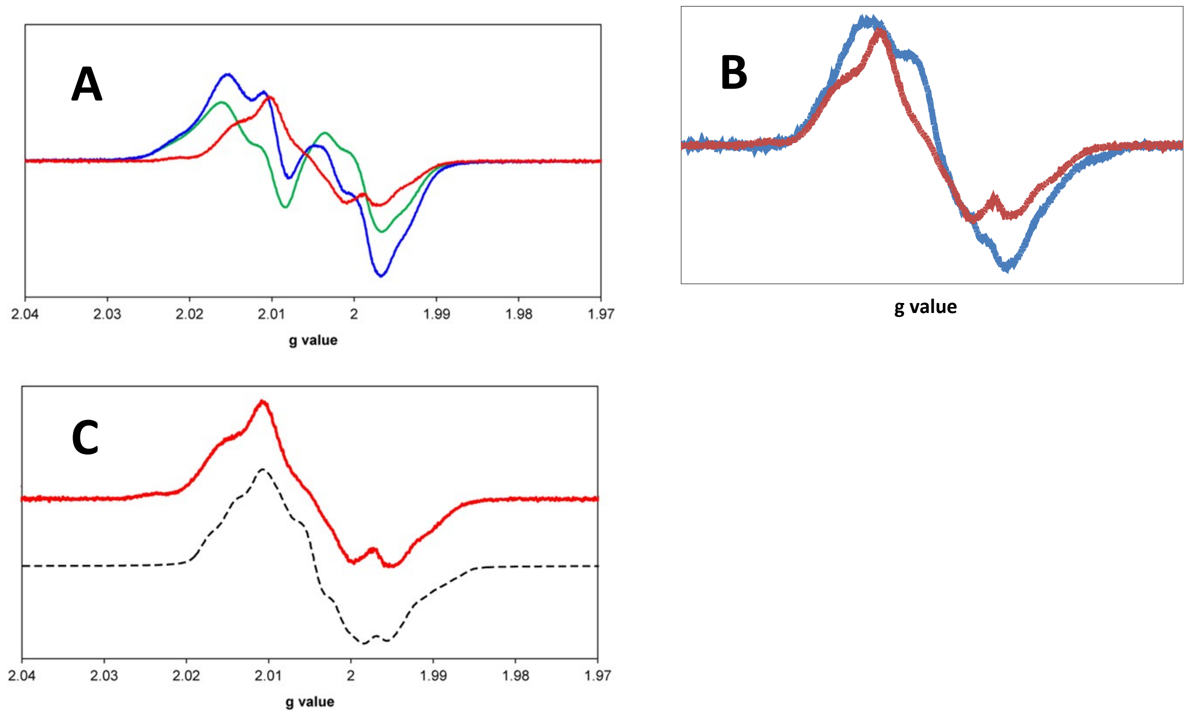

Figure S5. Reaction of 30 µM NH_2Y_{731}/R_{411}A-α2 with 30 µM wt-β2, 3 mM ATP and 1 mM CDP **A.** 9 GHz CW-EPR spectrum of the reaction mixture quenched after 30 s in a liquid isopentane bath. The reference Y_{122}•-β2 (green) spectrum was subtracted from the composite spectrum (blue) to yield the NH_2Y_{731}•/R_{411}A-α2 (red) spectrum. **B.** Subtracted NH_2Y_{731}•/R_{411}A-α2 spectrum (red, A) compared to NH_2Y_{731}•-α2 (blue) spectrum as reported previously.[8] **C.** NH_2Y_{731}•/R_{411}A-α2 (red) spectrum is shown with the corresponding simulation (black dashed line).

Figure S6: Conformations of $NH_2Y\bullet$ side chain. A) The ring dihedral $\theta_{C\beta}$ is defined as C_β-C_α-C_1-C_6 on an isolated $NH_2Y\bullet$ DFT model (**B**). The DFT model of the isolated $NH_2Y\bullet$ **(C)** The ring dihedral $\theta_{C\beta}$ was scanned in ~10 degree steps here illustrated by a few angles. (**D**) The energy in Hartree is plotted against the ring dihedral. The global energy minimum was found for $\theta_{C\beta}$ = 80 ± 15°. The g_x and g_y value as a function of dihedral angle is show in (**E**) Δg_x and Δg_y over the dihedral are 0.15 and 0.2 ppt, respectively.

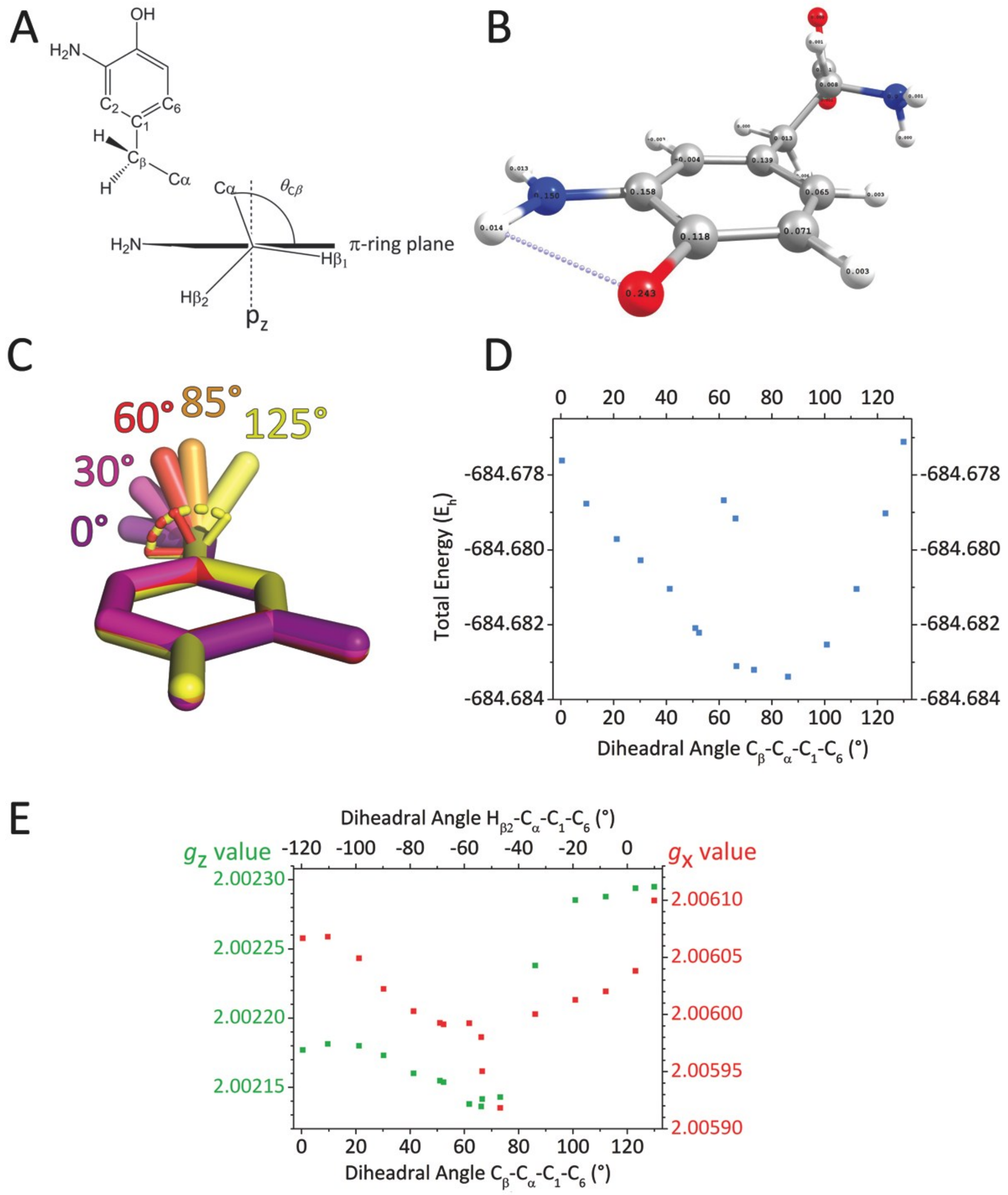

Table S2: Sample *xyz* coordinates (Å) of an optimized model.

	**Model 86°:** 25 Atoms (total energy = -684.6833912734 E_H)			
#		***x***	***y***	***z***
1	O	-4.163485	0.303454	1.673049
2	O	3.624982	-1.429799	-0.189077
3	O	3.770318	0.002656	-1.907949
4	N	-3.440707	2.025212	-0.283878
5	N	1.044058	-0.363036	-2.393481
6	C	3.098552	-0.642671	-1.136607
7	C	1.583085	-0.610137	-1.065146
8	C	1.213722	0.441707	0.033015
9	C	-0.233364	0.411725	0.436145
10	C	-1.161447	1.250598	-0.146041
11	C	-2.511009	1.226629	0.253093
12	C	-2.959725	0.304370	1.309875
13	C	-1.953257	-0.549783	1.875616
14	C	-0.655500	-0.496605	1.454359
15	H	1.828246	0.239449	0.912866
16	H	1.484340	1.429077	-0.345757
17	H	-0.857412	1.958628	-0.907798
18	H	-2.260704	-1.228676	2.660800
19	H	0.084067	-1.141913	1.912428

20	H	1.244501	-1.588842	-0.723805
21	H	1.415334	0.503376	-2.768275
22	H	0.035510	-0.267484	-2.353777
23	H	4.592891	-1.366406	-0.225223
24	H	-3.221329	2.70263	-0.994321
25	H	-4.381511	1.982405	0.076436

Figure S7: Comparison of simulation vs. experimental data for the 94 GHz EPR spectrum of ND_2Y_{731}•/R_{411}A-*α*.

We compared the EPR simulation with experimental spectra via a plot of residuals to show the quality of the simulation that takes into account only one radical species. There is no indication from the residuals for more radical species. The subtle differences in the line shape intensities between experiment and simulation can occur due to relaxation effects during spin echo detection, which are not considered in the simulation.

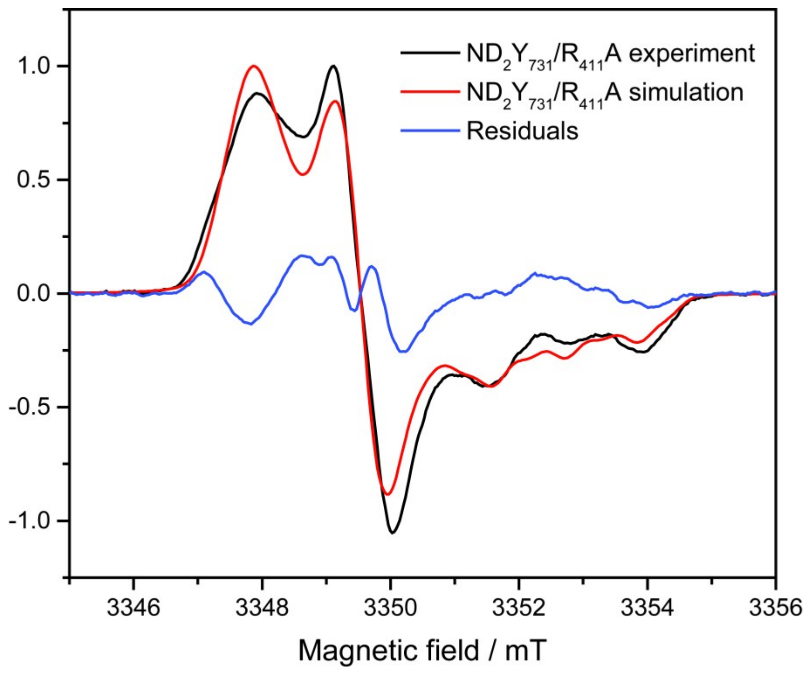

Figure S8: PELDOR excitation of ND_2Y_{731}•/R_{411}A in PELDOR at 20 K and 34 GHz. Top) Echo-detected EPR spectrum of ND_2Y_{731}•/R_{411}A-α after reaction with wt-β2, ATP and CDP in deuterated buffer containing 10% glycerol-(OD)$_3$ is a composite of Y_{122}•-β (dash-dotted black line) and ND_2Y_{731}•-α (dotted black line). Detect (D) and pump (P) excitation band widths for each PELDOR experimental setup used in this work are approximated with blue and purple rectangles, respectively. The width of the rectangles is $\Delta\nu = 1/t_p$ which is approximately the pulse width. Three sets of PELDOR measurements were enough to cover almost the whole echo-detected EPR spectrum of ND_2Y_{731}•/R_{411}A-α. Detect/pump π pulse lengths for D_1, D_2 and D_3 were 30 ns/12 ns, 30 ns/12 ns and 20 ns/14 ns, respectively. **Bottom, Left)** PELDOR time traces from three sets of PELDOR measurements (D_1, D_2, D_3 in blue) and their sum ($D_1 + D_2 + D_3$ in purple). **Bottom, Right)** Corresponding Fourier transformations are shown on the bottom, right side. The Pake pattern obtained from the summed PELDOR trace (solid purple line) almost matches the expected, ideal Pake pattern (solid red line) of the dipolar spectrum except for some intensity missing, as highlighted with black arrows.

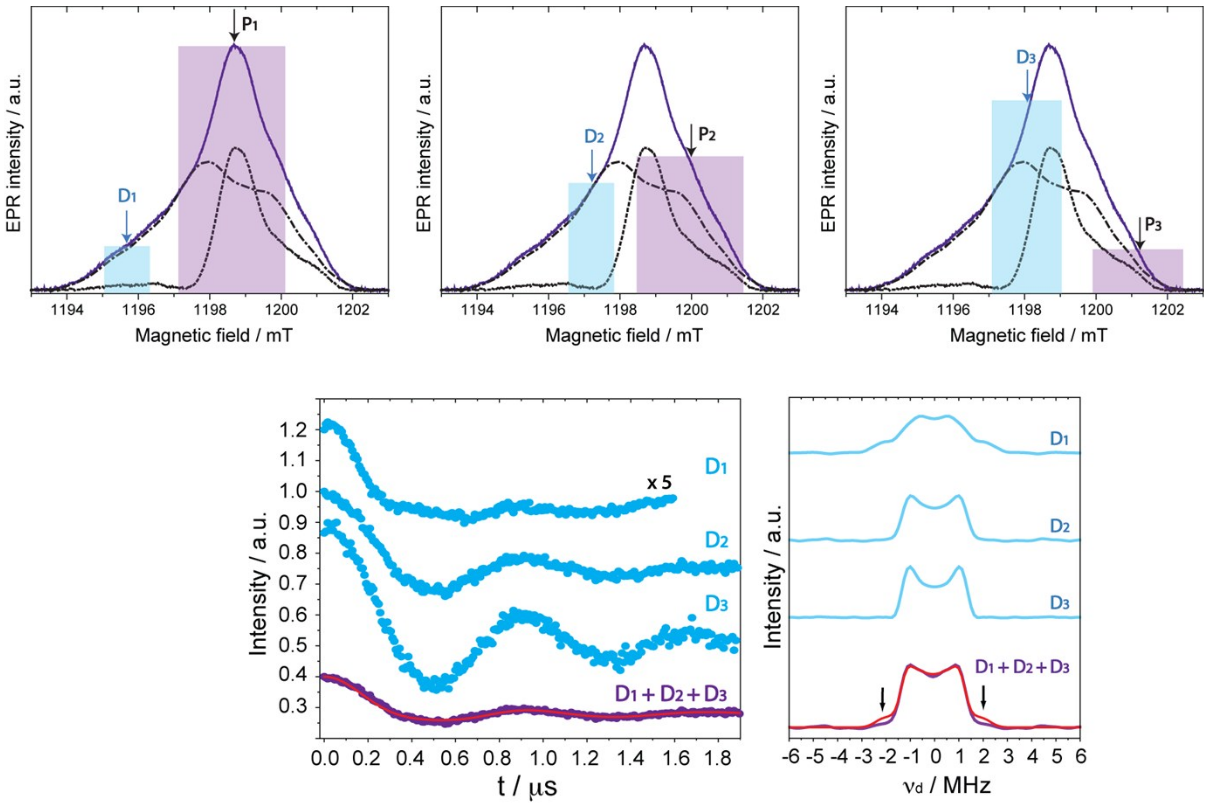

Figure S9: PELDOR measurement with ND_2Y_{731}•/R_{411}A-α at 50 K. Left) Refocused-echo spectrum of ND_2Y_{731}•/R_{411}A-α after reaction with wt-β2, ATP and CDP in deuterated buffer containing 10 % glycerol-$(OD)_3$. Y_{122}•-β (solid green line) is shown for comparison. Experimental conditions: $\pi/2$ = 6 ns, τ = 280 ns, shot repetition time = 20 ms, shots/point = 50, number of scans = 1, T = 50 K. Detect (D) and pump (P) excitation bandwidths are shown with blue and purple rectangles, respectively. ND_2Y_{731}•-α is the detected and Y_{122}•-β is pumped with this setup at 50 K. **Middle)** Background- and phase-corrected, normalized PELDOR time trace measured at 50 K and 34 GHz. Detect and pump π pulse lengths were 12 ns and 28 ns, respectively. The frequency difference between detect and pump positions was 80 MHz. The fit is overlaid in solid red line. Fitting was conducted by using DeerAnalysis2015.[9] **Right)** Resulting distance distribution. The width of the distribution in the PELDOR measurements is given as the half-peak width in the distance distribution. Asterisks indicate the artifacts attributed to the analysis procedure.

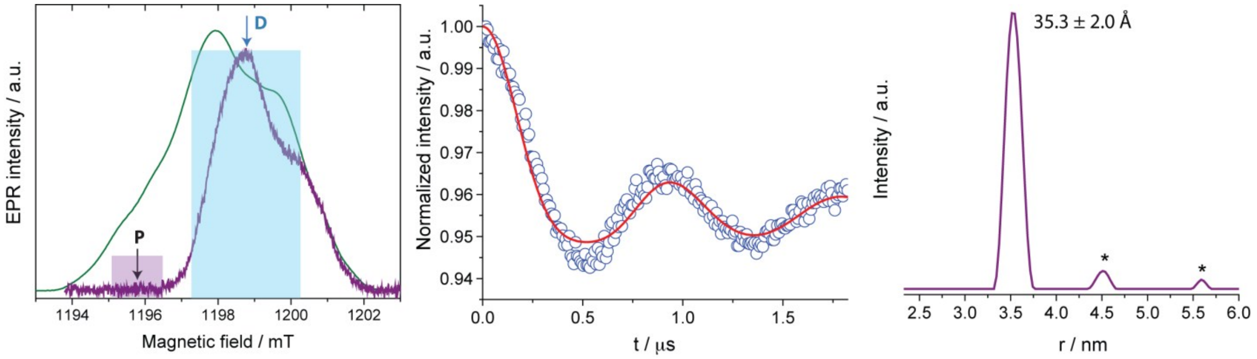

Figure S10: Examination of another possible conformation of ND_2Y_{731}•/$R_{411}A$-α

We have also examined diagonal distances for other possible conformations of NH_2Y_{731}, in which the amino group of NH_2Y_{731} is moved to occupy the vacancy created by the removal of the arginine side chain. We have aligned the $Y_{731}NH_2Y$-α structure (2XO5) with the *E. coli* $\alpha\beta$ docking model using PyMOL, which first performs a sequence alignment and then aligns the structures to minimize the root mean square deviation between the structures (see the figure below). In these alignments the rotated NH_2Y_{731} residue and R411A are shown in blue. In (A) only the ring of NH_2Y_{731} is rotated towards the residue 411. In (B) also the backbone of NH_2Y_{731} is moved so that the amino group can occupy the vacant hole left by removal of arginine side chain. The distances shown are between the phenolic oxygens of NH_2Y_{731} and Y_{122}. As shown in the figure, the distances for (A) and (B) do not reproduce the experimental distance of 3.5 nm and we could rule out these conformations.

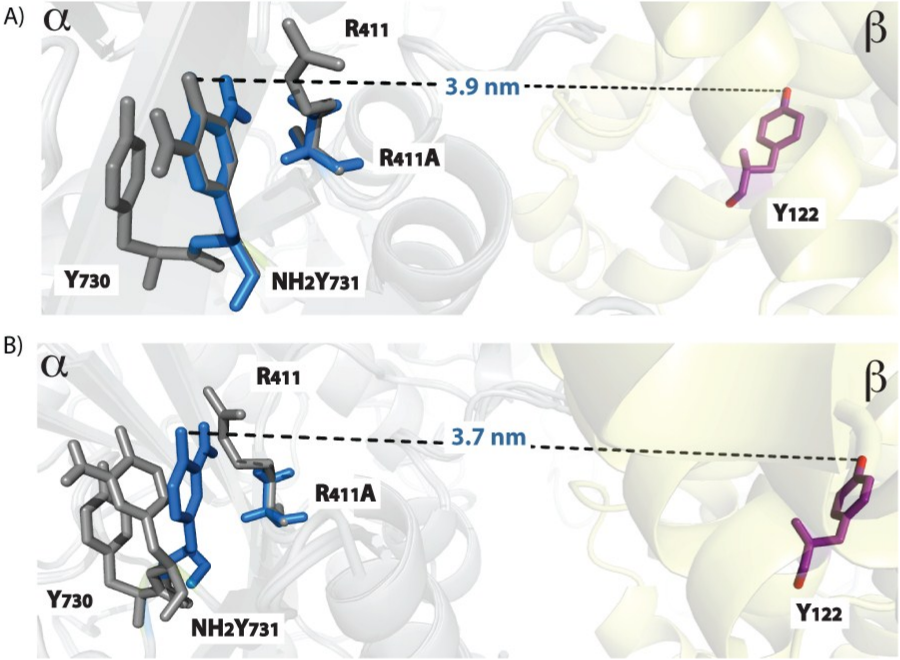

References

1. E. C. Minnihan, M. R. Seyedsayamdost, U. Uhlin and J. Stubbe, *Journal of the American Chemical Society*, 2011, **133**, 9430-9440.
2. T. U. Nick, W. Lee, S. Koßmann, F. Neese, J. Stubbe and M. Bennati, *Journal of the American Chemical Society*, 2015, **137**, 289-298.
3. I. Climent, B. M. Sjöberg and C. Y. Huang, *Biochemistry*, 1992, **31**, 4801-4807.
4. E. C. Minnihan, N. Ando, E. J. Brignole, L. Olshansky, J. Chittuluru, F. J. Asturias, C. L. Drennan, D. G. Nocera and J. Stubbe, *Proceedings of the National Academy of Sciences of the United States of America*, 2013, **110**, 3835-3840.
5. S. P. Salowe, M. A. Ator and J. Stubbe, *Biochemistry*, 1987, **26**, 3408-3416.
6. S. P. Salowe and J. Stubbe, *Journal of Bacteriology*, 1986, **165**, 363-366.
7. G. Palmer, *Journal*, 1967, **10**, 594-609.
8. M. R. Seyedsayamdost, J. Xie, C. T. Y. Chan, P. G. Schultz and J. Stubbe, *Journal of the American Chemical Society*, 2007, **129**, 15060-15071.
9. G. Jeschke, V. Chechik, P. Ionita, A. Godt, H. Zimmermann, J. Banham, C. R. Timmel, D. Hilger and H. Jung, *Applied Magnetic Resonance*, 2006, **30**, 473-498.
